# Hyperbaric oxygen for stroke treatment

**DOI:** 10.1186/2045-9912-1-4

**Published:** 2011-04-27

**Authors:** James Toole

**Affiliations:** 1 Department of Neurology, Wake Forest University School of Medicine Medical Center Boulevard, Winston-Salem, NC 27157-1002, USA

## Abstract

This is an editorial for the inauguration of the Medical Gas Research and addresses a particular issue of using hyperbaric oxygen for stroke treatment.

## Editorial

Stroke is one of the most expensive as well as the third most common cause of disability and death in industrialized nations. In the US its prevalence is 5.8 million causing about $73.7 billion dollars for the Year 2010. Because of increasing life-span, these numbers are likely to increase even more. Consequentially, prevention and optimal management of those suffering will increase health care expenditures. When I became a physician, there was no effort to prevent stroke or to identify transient ischemic attacks (TIA). Consequently the Princeton Conferences on stroke prevention and treatment were founded. Having attended these conferences intermittently since 1961, I suspect I am now a senior medical person. Few are aware that Irving S. Wright founded this conference. He was a cardiologist who introduced heparin to the United States from Sweden by initiating the First Prospective Randomized Trial for therapy of stroke in which I was a participant. It was he along with Mrs. Mary Lasker, who developed the concept for the Princeton Conferences in order to influence national policy regarding research in heart disease and stroke. Mrs. Lasker explained for me that one of her friends experienced a stroke and was told there was no treatment other than bed rest. Together they decided to change this sorry state of affairs: Mrs. Lasker supplied funds and Dr. Wright the impetus for inviting colleagues from many disciplines to Princeton, New Jersey in the bitter cold of January to develop a plan for national action for stroke prevention and treatment. Thereafter, Mrs. Lasker and her friend, Florence Mahoney, devoted their efforts to this neglected field and urged President Lyndon Johnson to change the name of the National Institute for Neurological Diseases and Blindness to include the word "stroke" so that a national effort could be initiated in this in this neglected field.

The above is a preamble for my belief that innovations in therapy for stroke have been neglected even though many trials have been performed - and one is hyperbaric oxygen therapy for acute stroke and rehabilitation. Hyperbaric oxygen for stroke is not a new idea! Why has it been so difficult to convince my colleagues in healthcare that oxygen for those who are with acute stroke deserves a randomized and prospective clinical trial?

The administration of oxygen to people who suffer cardiac events or hypoxic from respiratory disorders are standard. We know that the brain requires more oxygen than any other organ in the body; therefore, defies logic to deny hyperbaric oxygen. Why is it that cardiac disorders are routinely placed in oxygen and stroke victims are not? Of all the organs in the body, the brain requires the most oxygen and is the most vulnerable to hypoxia. Consequently, it is not logical to limit its use to cardio-pulmonary disorders and wound healing.

In here, I would like to show a letter from Edward Teller from the Hoover Institution on War, Revolution and Peace, from the Stanford University. Edward Teller was one of the physicists who participated in the Manhattan Project and later on became known as the Father of the Hydrogen Bomb (http://www.u-s-history.com).

December 5, 2000

James Toole, M.D.

Broman Gray School of Medicine

Medical Center Boulevard

Winston-Salem, North Carolina 27151

Dear Dr. Toole,

Dr. Neubauer visited me on his return from Australia. He has informed me of your many accolades and the substantial role you have played in the development of the field of neurology. I want to take this opportunity to tell you how pleased I am that you, with an open mind, are looking into the potential use of hyperbaric oxygenation for treatment of the acute stroke. I personally have had experience with this modality for the past several years. I had a stroke approximately five years ago and the local hospital would not treat me. Dr. Neubauer made arrangements for me to have a home chamber and since that time I have taken several treatments a week. Although I may well have recovered and done very well without the supplemental high dose oxygen, my attendants stated very clearly that this has made somewhat of a positive change in my ability to continue an active work and lifestyle. There certainly should be a place for the use of hyperbaric oxygenation in certain disease states and especially in the field of neurology.

If is possible for you, send me quantitative statistically significant statements about hyperbaric work in neurology, particularly your own work. I should be greatly interested.

Again, I congratulate you on your far-sightedness and I trust that someday hyperbaric oxygenation may become an accepted therapy.

Sincerely,

Edward Teller

ET: Imn

Cc: Dr. Neubauer

For inexplicable reasons, hyperbaric oxygen therapy has been pushed aside and categorized as "unproved" and no one sees fit to put it to the test with a properly designed prospective randomized trial.

